# Aromatic Potential and Bioactivity of Cork Stoppers and Cork By-Products

**DOI:** 10.3390/foods9020133

**Published:** 2020-01-28

**Authors:** Ana Maria Mislata, Miquel Puxeu, Raul Ferrer-Gallego

**Affiliations:** 1 Centro Tecnológico del Vino—VITEC, Carretera de Porrera Km. 1, 43730 Falset, Spain; anamaria.mislata@vitec.wine (A.M.M.); miquel.puxeu@vitec.wine (M.P.); 2 Sensometria Instrumental (i-Sens), Department of Analytical Chemistry and Organic Chemistry, Universitat Rovira i Virgili, 43007 Tarragona, Spain

**Keywords:** cork, volatile compounds, antioxidant activity, polyphenols, aroma, waste

## Abstract

The characterization of natural waste sources is the first step on the reutilization process, circular economy, and global sustainability. In this work, the aromatic composition and bioactive compounds related to beneficial health effects from cork stoppers and cork by-products were assessed in order to add value to these wastes. Twenty-three aromatic compounds with industrial interest were quantified by gas chromatography coupled mass spectrometry GC–MS in both samples. Vanillins and volatile phenols were the most abundant aromatic families. Other aromatic compounds, such as aldehydes, lactones, terpenols, and alcohols, were also determined. Furthermore, the phenolic composition and the antioxidant activity were also evaluated. Overall, extracts showed high aromatic and antioxidant potential to be further used in different industrial fields. The recovery of these valuable compounds from cork stoppers and cork by-products helps to reuse them in agricultural, cosmetic, pharmaceutical, or food industries.

## 1. Introduction

Nowadays, a key issue in any field of research is the fight to curb climate change. This environmental awareness is of great importance in every society in order to achieve a sustainable environment considering the current human actions. The re-use and material recycling is a priority for waste management in the European Union (Directive 2008/98/EC on Waste). The characterization of natural waste sources is the first step on the reutilization process and the global sustainability. Bioactive compounds related to beneficial health effects and aromatics from by-products can be explored by food, agricultural, cosmetic, or pharmaceutical industries. Fragments, granulates, and powder from cork represent a large waste stream from cork processing [1,2]. They have been commonly used as combustion fuel, although they have also been employed in agriculture. Composted residues from cork were used as plant growth media to suppress plant diseases [3], and hydrological properties of substrates based on industrial cork residue have also been reported [4]. Recently, their adsorption properties, such as fining agent in wines, were stated [5], and some works showed that cork wastes are cost-effective and green alternatives to the retention of contaminants from water [6,7,8,9]. However, to the best of our knowledge, few works were reported regarding their revalorization for the food industry. According to statistical reports of the International Organization of Vine and Wine (OIV), the annual world wine production is around 275 million hectoliters (292 MhL in 2018) [10], and 90% of bottle wines are stopped by corks. In cork stoppers production (about 300 thousand tons of cork are produced annually), the cork waste represents around 25% of the raw material. Different cork wastes can be found depending on their characteristics, density, moisture, granulometry, size, ash content, and tannin concentration [1,11,12].

It is well known that the phenolic composition of plants is related to antioxidative, anticarcinogenic, and antitumor biological activities [13,14]. Specially, polyphenols from *Quercus suber* L. have been associated with beneficial health effects linked to hydrolysable tannins and phenolic compounds with low molecular weight [15]. Recent research valorized cork powder and granulates from a phenolic point of view [12], however, little research focused on the recovery of valuable aromatic compounds. On the other hand, a great number of works are related to the off-flavors or cork-taint compounds in wine [16,17,18,19]. However, the characterization of the positive aromas of cork stoppers and cork by-products is scarce. In this work, the aromatic composition of different wine cork stoppers and granulates was determined, enhancing the added value of their re-use in other industrial fields. The tannin concentration and the antioxidant activity of the cork extracts were also compared and discussed.

## 2. Materials and Methods

### 2.1. Samples and Extraction

**Figure 1:**
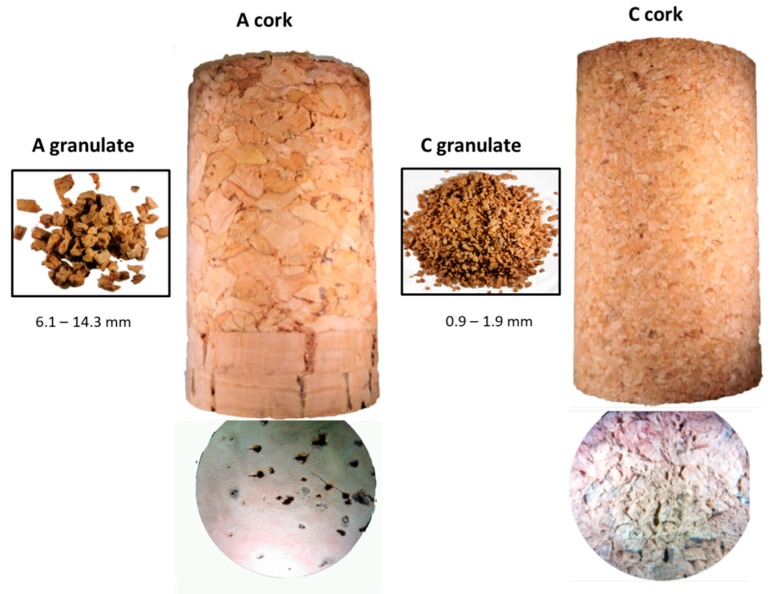
Representative image of the studied granulates and cork stoppers (A, high particle size and C, low particle size).

Different cork stoppers and their respective cork granulate (A, B, and C), from which the corks were made, were evaluated in this study. The granulate cork size ranged from 6.1 to 14.3 mm for A samples (high size), from 1.6 to 4.4 mm (medium size) for B samples, and from 0.85 to 1.9 mm (low size) for C samples (Figure 1 and Table S1). The cork stoppers dimensions were 30 × 50 mm for all samples. To determine the migration of the phenolic compounds from granulates and cork samples to model solution, a liquid–liquid extraction with ethyl acetate was made according to Azevedo et al. [20]. Briefly, 30 g of each sample (cork stoppers and granulates) was weighted in glass jars and macerated in 1 L of hydro alcoholic solution. The model solution contained 13% of ethanol, 5 g/L of tartaric acid, and the pH was adjusted to 3.7 with sodium hydroxide (1 *N*). Solutions were stored at controlled temperature (20–22 °C) until the analyses were performed. Different times of maceration were evaluated in this study (3, 5, and 15 days).

### 2.2. Reagents

Protocatechuic acid and gallic acid were purchased from Sigma-Aldrich (Steinheim, Germany). Folin–Ciocalteu reagent, sodium carbonate anhydrous, ethyl alcohol 96%, and acetic acid glacial were purchased from PanReac AppliChem, Barcelona, Spain. All the aromatic standards were supplied by Sigma (Sigma Aldrich, Merck Life Science, Barcelona, Spain). Vanillin 99%, guaicol 98%, eugenol 98%, benzaldehyde ≥ 99%, nonenal 97%, phenylacetaldehyde ≥ 90%, phenylethyl alcohol ≥ 98%, benzyl alcohol ≥ 99%, camphor 96%, borneol 97%, 4-terpineol ≥ 96%, α-terpineol 90%, γ-nonalactone ≥ 98%, nonanoic acid ≥ 97%, vanillic acid ≥ 97%, octanoic acid ≥ 99%, dodecanoic acid 98%, benceneacetic acid ≥ 99%, furfural 99%. Dichlorometane anhydrous 99.8%, pentane anhydrous ≥ 99%, acetonitrile anhydrous ≥ 99.8%, phosphate buffered saline (PBS), potassium peroxodisulfate ≥ 99%, 6-hidroxy-2,5,7,8-tetramethyl-chromane-2-carboxylic acid 97% (Trolox), and 2,2′-Azino-bis(3-ethylbenzothiazoline-6-sulfonic acid) diammonium salt (ABTS) were also purchased from Sigma Aldrich, Merck Life Science, Barcelona, Spain.

### 2.3. GC–MS Analysis

A volume of 100 µL of 2-octanol, as internal standard, was added to 100 mL of extract. Afterward, samples were separated by a SPE (solid phase extraction) cartridge (Bond Elut ENV, 500 mg and 6 mL, Agilent Tech., Santa Clara, California, USA). The cartridges were previously conditioned with 5 mL of dichloromethane, 5 mL of ethanol, and 5 mL of hydroalcoholic solution (12%). Analytes were eluted with pentane-dichloromethane (50:50, *v*/*v*), then dried using a concentrator vacuum (Savant™ SPD131DDA, Thermo Fisher Scientific, Barcelona, Spain). Finally, samples were redissolved in 200 μL of dichloromethane.

GC analysis was performed on a GC 7890A (Agilent Tech., Santa Clara, California) system equipped with a mass spectrometer 5975C inert MSD (with Triple-Axis Detector). The column was a DB-5 (30 m × 0.25 mm × 0.25 μm, Agilent Tech.). A constant flow of 2.1 mL/min of He was used as carrier gas. Five microliters of sample was injected in splitless mode with 17.33 psi pressure (septum purge flow 15 mL/min and splitless time 1 min.). The injector temperature was maintained at 225 °C for 1 min and then heated up to 250 °C at 5 °C/min. The temperature of the oven (40 °C) was maintained for 1 min and then increased up to 260 °C at (20 °C/min.) for 25 min. The mass spectrometer operated at 70 eV (electron ionization) modes. The analysis was performed in Scan mode (m/z 10−1000). The compounds were identified by retention times and mass fragments, to compare with those of pure standard compounds. The quantification was carried out using internal standard patterns.

### 2.4. Total Phenolic Content

The phenolic composition was determined by the Folin–Ciocalteu assay and HPLC–DAD/MS analysis. Total phenols (TP) were determined using the Folin–Ciocalteu assay [21] with some modifications. Briefly, 100 μL of sample, 500 μL of Folin–Ciocalteu reagent, and 2 mL of a solution of sodium carbonate (20% *w*/*v*) were mixed, final volume 10 mL with water. The solution was stocked for 30 min for the reaction to take place and stabilize and finally, the absorbance was measured at 750 nm.

Chromatographic analyses were carried out in an Agilent 1200 series (Agilent Technologies, Palo Alto, CA, USA) coupled with DAD and MS detectors. A volume of 50 mL of each extract was washed 3 times with 20 mL of ethyl acetate. The organic phases were pooled and evaporated, re-dissolved in 1 mL of water/methanol (50:50) and then identified and quantified by HPLC–DAD/MS. A Zorbax Eclipse Plus C18 column (3.5 μm, 150 × 4.6 mm) was used. The chromatographic conditions were used according to Azevedo et al. (2014) [20]. Briefly, solvent A was 0.1% of acetic acid in water, solvent B was acetic acid, acetonitrile, and water (1:20:79, *v*/*v*/*v*). The gradient was from 80% to 20% of solvent A over 55 min, from 20% to 10% of A from 55 to 70 min, and from 10% to 0% of A from 70 to 90 min. The flow rate was 0.3 mL/min, and the sample injection 20 μL. Gallic acid and protocatechuic acid were identified by the retention time and UV–VIS spectra. The compounds were quantified with phenolic standards using peak area data of resolved peaks at 280 nm. The corresponding calibration curves were made up for gallic acid (r^2^ = 0.999) and protocatechuic acid (*r*^2^ = 0.999). The identity of the phenolic compounds was confirmed by mass spectrometry. A TSQ Quantum™ Access MAX (Thermo Fisher Scientific, Waltham, MA USA) equipped with an HESI (Heated Electrospray Ionization) source which was operated in the negative ionization mode between *m/z* 80 and 800 was used. The HESI spray voltage was set at 3.5 kV, and the capillary temperature was maintained at 350 °C. Nitrogen was used for nebulization and desolvation (sheath gas 60 arb. and auxiliary gas 20 arb.). The vaporizer temperature was maintained at 350 °C. Argon was used as the collision gas for collision-induced dissociation.

### 2.5. Antioxidant Activity Assay

The ABTS method allows determining the antioxidant activity through the discoloration of the cationic ABTS^+^ radical produced by the oxidation of ABTS with potassium persulphate. This assay was performed according to the procedure described by Re. et al. (1999) [22] with slight modifications. A stock solution of 7 mM ABTS in water was prepared. To form the radical cation, a solution of potassium persulfate (2.45 mM) was prepared, using the ABTS stock solution as solvent. This solution was stored at 4 °C in the absence of light to complete the reaction. To prepare the working reagent, the solution was diluted with phosphate buffered saline (PBS) at pH 7.4, until an absorbance around 0.7 at 734 nm was obtained. Trolox or samples were added, and the decrease on the absorbance at 734 nm was measured, since the coloration disappears when the radical is reduced by antioxidants. Blank was made by adding 2 mL of reagent in a cuvette, and its absorbance was measured at 734 nm. Subsequently, 50 µL of diluted sample was added and vortexed for 30 s. After 4 min of incubation at room temperature, the absorbance was measured again at 734 nm, results were expressed in μmol/L of Trolox reagent. Samples were analyzed in several concentrations, making five dilutions of each sample by duplicate.

### 2.6. Statistics

Xlstat 2016.01 statistical software (Microsoft Ibèrica, Barcelona, Spain) add-on for Microsoft Excel package (Microsoft Ibèrica, Barcelona, Spain) was used for data processing. Significant differences between granulates and cork samples were determined by one-way ANOVA using the Tukey’s HSD (honestly significant difference) test, at 95% of confidence level.

## 3. Results and Discussion

### 3.1. Aromatic Characterization

A total of 23 aromatic compounds were determined in granulates and cork samples and grouped according their chemical structures. Vanillins and derivatives, volatile phenols, aldehydes, alcohols, terpenes, lactones, fatty acids, and furans were found. A lot of these volatiles are closely related to certain pleasant aromatic descriptors. Table 1 shows the minimum and maximum content of the individual compounds found. The most important compound in the analyzed samples was vanillin (up to 170 µg/g), and to a lesser extent 4-vinylguaicol (23 µg/g), acetovanillone (14 µg/g), and dodecanoic acid (6.3 µg/g). All these volatiles have very pleasant aromas, such as vanilla, coconut, or wood, highly used in culinary industry and cosmetics.

**Figure 2:**
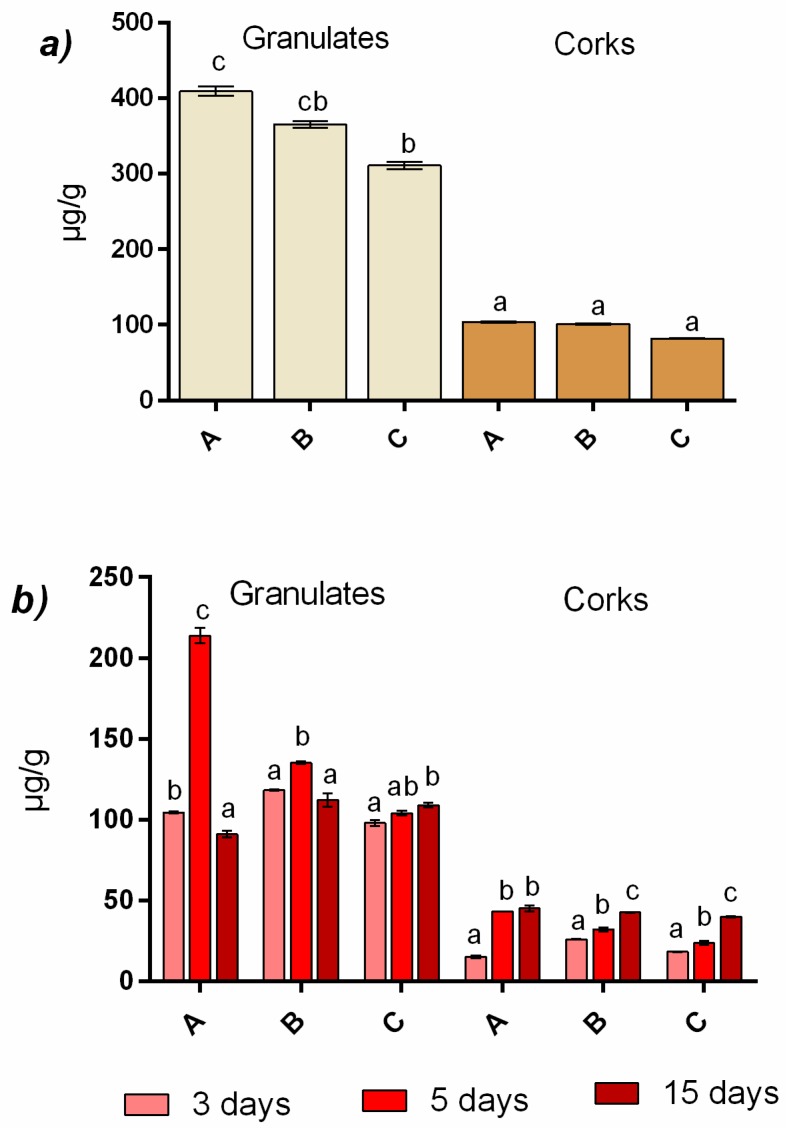
Total aromatic compounds (mean ± SD) of granulates and corks (**a**) and total aromatic compounds of granulates and corks at different time of extraction (**b**). Different particle size from cork stoppers and their respective granulates (A; high, B; medium, C; small). Different letters indicate significant differences, *p* < 0.05.

**Figure 3:**
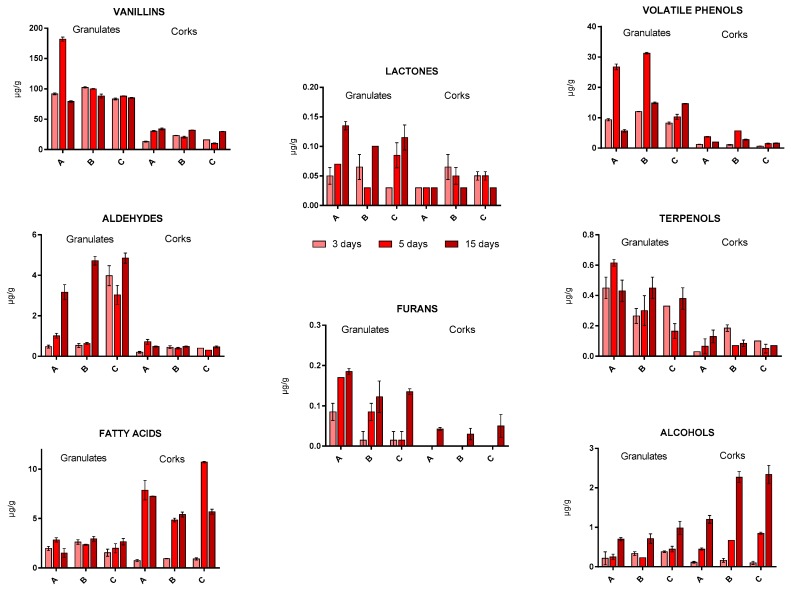
Composition per family of the studied aromatic compounds. Different particle size from cork stoppers and their respective granulates (A; high, B; medium, C; small).

Figure 2a shows the sum of the total aromatic content of granulates and corks. In this figure, we can observe that the granulate samples extracted about 75% more than the cork stopper samples. Curiously, the highest amount of aromas were extracted in A granulate, corresponding to the largest particle size, followed by B granulate (medium size), and finally C granulate (smallest size). This could be due to the differences on the weight-volume relationship of granulates and their porosity, since the natural cork is a heterogeneous material with structural differences [23]. In this case, the smaller size of particles did not contribute to an increase of the extraction, likely because of differences in the volume of lenticels and dense matter [24]. Regarding cork stoppers, significant differences were not observed, A and B extracted a similar amount of aromas, slightly higher than that of C. Granulate samples (A and B) obtained the highest concentrations of total aromas after 5 days of maceration (Figure 2b), while for corks the maximum content was generally obtained at the end of the assay (after 15 days of extraction). In granulate samples, the highest amount of aromatic compounds may be reached earlier than in cork samples, likely because the characteristics of the samples and size facilitate the extraction of volatiles. In the studied samples, it seems that the bigger the granulates, the faster the extraction rate of the volatiles. The A granulates that correspond with the highest granulate size, mean value around 9.2 mm, obtained the highest amount of volatiles reaching levels over 200 µg/g. This increment in A granulate at 5 days is mainly due to a high extraction of vanillins and volatile phenols, and to a lesser extent to terpenols and fatty acids (Figure 3).

Considering the time of extraction in cork stoppers, only in the case of A corks was the maximum value of volatiles reached in 5 days of maceration, since for B and C corks, the maximum values were reached after 15 days of maceration (Figure 2).

Figure 3 shows the total aromatic composition of the extracts per studied family. Vanillins are the most important family. In this study, this compound reached values from 9.3 µg/g (in C corks) to 167.9 µg/g (in A granulates, Table S2). Therefore, vanillin has a great impact on the overall aroma of cork extracts. This compound is very characteristic for providing a very intense and pleasant aroma with sweet and floral notes, which is currently used in many different industrial fields, such as in the production of fragrances, in food use as in baking [25], or in cosmetics [11]. In addition, as was seen in other studies [26,27], vanillin is a natural bioactive component present in corks, which showed an important antioxidant activity. Another important aromatic family of compounds studied in the food industry were volatile phenols [28,29]. As shown in Figure 3, the maximum content of these aromatic compounds was extracted after 5 days of maceration in A and B granulates. This pattern seems to be also reproduced in A and B cork samples. It may be due to the high concentration of vinylguaiacol (values up to 23 µg/g) and, to a lesser extent, to guaiacol content (5 µg/g). Both compounds are characteristic for having aromas of spicy notes, specifically clove, wood, and smoked [30,31].

Regarding aldehydes, it was observed that the granulates extracted the highest concentrations after 15 days of maceration with values up to five times higher with respect to the corks. The main compounds were phenylacetaldehyde with maximum values of 4.5 µg/g and nonenal with values of 0.47 µg/g. These compounds are characteristic for providing fresh and intense aromas even at low concentrations, such as green grass, citrus, and wax [32]. It should be noted that the odor threshold for phenylacetaldehyde in hydroalcoholic solution was established in 5 µg/L [33], and the maximum content found in this work corresponded to 135 µg/L (30 g of cork in 1 L). In this way, the maximum nonenal amount in these samples was 14.1 µg/L higher than the established odor threshold determined in water (0.065 µg/L) and in hydroalcoholic solution (0.17 µg/L) [34].

Lactones exhibited a behavior similar to that of aldehydes. The aroma of lactones is of interest for commercial aromatization of food [35]. Here, γ-nonalactone is the compound that represents this family. This compound is better extracted from granulates than from corks after 15 days of maceration. Despite presenting low concentrations (0.11 µg/g) in the extracts, its concentration is higher than the odor threshold in water (0.03 µg/g) [35]. This is a characteristic compound for providing pleasant aromatic descriptors, such as coconut, peach, and sweet cream butter notes [36,37]. This aroma is commonly used in the development of cosmetics and fragrances. In addition, they could also be used in the food industry as an essence in the preparation of cakes, sweets, candies, and ice cream, as margarine or aroma [36].

Similarly, the terpenols had a higher concentration in the macerates of granulates than in corks, especially after 15 days of extraction. The contribution of the individual terpenols seems to be similar, since little differences were observed among them. Their concentrations ranged from 0.05 (A cork after 3 days) to 0.62 µg/g (A granulate after 5 days) (Table S2). Terpenes are aromatic compounds commonly synthesized in plants, trees, and vegetables. They are usually the main constituents of essential oils of most plants, offering a wide variety of pleasant scents, from flowery to fruity, to woody, or balsamic notes [38]. Hence, cork has a wide range of terpenoid variety within the family of terpenols formed in this study by the following determined compounds: camphor, borneol, α-terpineol, and 4-terpineol. All of them have interesting aromatic descriptors such as mint, pine, spices, and flowers, respectively. In addition, today they are used in cosmetics, especially in the elaboration of anti-aging creams, because they possess bioactive properties [39]. They may act as elastase inhibitors, preventing the structural degradation of elastin fibers in the dermal matrix [2,40,41], and play an important role as constituents of flavors for spicing foods, sweets, beverages, and baked foods [42]. Furfural, an important aromatic additive in food and beverages [43], also showed the highest concentrations after 15 days of maceration, being higher in granulates (5.7 µg/L) than in corks (1.5 µg/L). The odor threshold of this compounds is also lower (1 µg/L) [44] than the concentrations found in the studied samples, as occurred in other compounds.

On the other hand, alcohols and fatty acids showed higher concentrations in macerated cork stoppers than in granulates (Figure 3). With regard to alcohols, granulates and corks showed a large increase in concentration after 15 days of maceration, reaching values of up to 0.98 µg/g and 2.32 µg/g, respectively. Among them, the phenylethyl alcohol stands out for presenting the highest concentrations (Table S2), and for having interesting aromatic descriptors, such as flowers. For this reason, this compound is commonly used in the production of perfumes as well as flavoring in the food industry. It is also used in the cosmetic industry for the preparation of creams and soaps, acting as a preservative due to its stability in basic conditions. Another important property of this compound is its antimicrobial activity [45]. Fatty acids showed a large increase in concentration after 5 and 15 days of maceration in cork stoppers. This family reached a value of 10.74 µg/g. It should be noted that their concentrations were three times higher than in the case of granulates. The main fatty acids determined were vanillic, bezenacetic, decanoic, and octanoic, highlighting the latter for having the highest concentrations. All of them contributed to providing very pleasant aromatic notes such as vanilla, honey, lactic, and coconut, respectively.

The great increase in the concentration of phenylethyl alcohol and octanoic acid in the macerates of corks with respect to granulates could be due to the presence of this compound in glues used for the manufacture of agglomerated corks [46]. Their use is due to their aromatic, antimicrobial, and antibiotic properties.

In summary, the families studied provide the aromatic profile of the granules and cork stoppers, which are rich in very pleasant aromas at the sensory level. This aromatic composition extracted from cork could have a second shelf life in different types of industries. On the one hand, the cosmetic and pharmaceutical industries could use these extracted compounds as ingredients in the manufacture of products such as sunscreens, wrinkle products, fragrances, or even soaps. On the other, they could be also used in processed food as flavoring additive, and it should be noted their bioactive properties and beneficial health effects, such as antioxidant and antimicrobial activities.

### 3.2. Phenolic Composition

**Figure 4:**
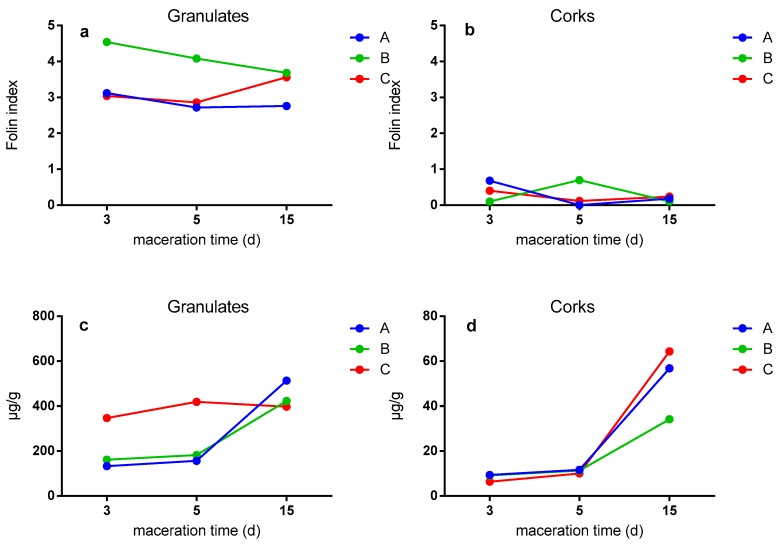
Folin–Ciocalteu index (**a** and **b**) and phenolic content determined by HPLC (**c** and **d**; note difference in *y*-axis scales). Particle size from cork stoppers and their respective granulates, A; high, B; medium, C; small.

Many studies have used the Folin–Ciocalteu method to determine polyphenols in plant extracts [47,48]. The combination of HPLC–DAD/MS analysis with this methodology helps the determination of the compounds, from the individual to the polymeric polyphenols. Figure 4 shows the Folin–Ciocalteu index (Figure 4a,b) and the content in the phenolic compounds in the studied cork samples determined by HPLC (Figure 4c,d).

In general, higher polyphenol content was obtained for granulates than for corks. A and B granulates obtained the highest value after three days of maceration (Figure 4a). As shown in Figure 4c,d, in general, a higher concentration of phenolics was extracted after 15 days of maceration. In the case of granulates, it is observed that A and B granulates had concentrations above 150 µg/g after 3 and 5 days of maceration, and increased to concentrations around 500 µg/g after 15 days. The highest concentration was obtained in A granulate (513.5 µg/g), with larger particle size. In C granulate (smaller size), the extraction was more constant, around 350–400 µg/g for all maceration times. After 15 days of maceration, the phenolic composition in cork stoppers increased in all cases from 10 µg/g to 65 µg/gin in the C sample. Other authors observed differences in the phenolic extraction depending on the type of cork stopper, granulate, and powder [12,20,49]. Further research should be done to optimize time of extraction and methods. Furthermore, the use of new promising technologies may be interesting in order to optimize the process in a real scale [50].

Considering the individual content of the phenolic compounds, gallic acid obtained the highest concentrations with values between 60.6 and 180.9 µg/g, followed by protocatechuic acid with values between 41.3 and 161.6 µg/g, and finally the protocatechuic aldehyde with values between 26.3 and 118.6 µg/g. As stated in previous studies [51,52], gallic acid and protocatechuic acid are phenolic compounds with abundant presence in cork extractive fractions.

### 3.3. Antioxidant Activity

Previous studies have already shown that the cork has bioactive compounds with antioxidant activity [2,53]. Touati et al. studied cork extracts in methanol solution and water using the ABTS method, demonstrating its high antioxidant activity, and Fernandes et al. characterized the great antioxidant activity of cork extracts, which was directly related to the phenolic composition [15,54].

**Figure 5:**
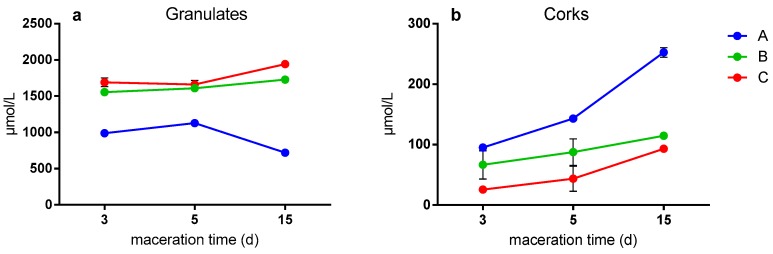
Antioxidant activity of granulates (**a**) and cork (**b**) samples determined by ABTS method (expressed in µmol/L of Trolox reagent), note difference in *y*-axis scales. Particle size from cork stoppers and their respective granulates, A; high, B; medium, C; small.

Figure 5 shows the antioxidant activity in μmol/L of Trolox of the granules and corks throughout the maceration time. In general, it is observed that granulate samples obtained higher antioxidant activity than corks (up to one hundred times). Figure 5A shows that B and C granulates had higher antioxidant activity compared with the larger A granulate. Likewise, the three types of granulates obtained similar antioxidant activity after all maceration times, as reported Azevedo and co-workers, who observed no significant differences in wine model solution at different times when bottling with different cork stoppers [20]. With respect to the corks (Figure 5B), in general, the same trend is observed in all types of corks. In this case, the antioxidant activity increases by increasing the maceration time. The differences observed between samples after 15 days of maceration may result from the higher amount of simpler phenolic compounds at this time (Figure 4d). The maceration of A cork (larger granulate) presented the highest concentration at all times, especially after 15 days of maceration. On the one hand, this trend is not explained from the obtained data, so this could be due to hydrolyzable tannins that are powerful antioxidant agents and were not determined in this study [15].

On the other hand, the high antioxidant capacity of the cork samples studied, especially in the case of granulates, could be directly related to phenolic acids, in particular the high concentrations of gallic acid and protocacatechuic acid [26,54]. In addition, this antioxidant capacity could also be due to the aromatic composition of the corks studied, especially to the high concentrations of vanillin (170 µg/g), which is considered a natural bioactive component of cork [15,55]. According to Azevedo et al., the amounts of gallic acid, protocatechuic acid, protocatechuic aldehyde, and vanillin in cork samples appear to be crucial for the antioxidant activity [20].

## 4. Conclusions

Overall, this work highlights valuable aromatic compounds (vanillins, volatile phenols, aldehydes, alcohols, terpenols, lactones, fatty acids, and furans) found in cork by-products and cork stoppers to be reused as flavoring agents and antioxidants in the food industry. The most important family of compounds was vanillins with a high content of vanillin and acetovanillone, and 4-vinylguaicol was the most abundant volatile phenol. Furthermore, the phenolic composition of corks and their by-products may provide interesting antioxidant properties with increasing interest on the industry for their health benefits. Granulates showed higher potential than corks, however, corks used daily (e.g., wine stoppers) have to be considered for this purpose. Further research should be done in order to optimize waste management and extraction procedures, among others.

## Figures and Tables

**Table 1:** Aromatic compounds, families, descriptors, and minimum and maximum content found in the studied granulates and cork macerates.

Aromatic Compound	Aromatic Descriptor	Content (µg/g)
**Vainillins**		
Vanillin	vanilla	9−170
Acetovanillone	vanilla	0.6−14
**Volatile phenols**		
Guaicol	wood, smoked, sweet, medicine	0.03−5.0
4-vinylguaicol	wood, spice cloves, curry	0.5−23
Eugenol	spice cloves, honey	0.01−0.3
Isoeugenol	carmination	0.06−2.4
Cerulignol	spicy	0.04−2.2
**Aldehydes**		
Benzaldehyde	almonds, sweet, caramel	0.02−0.21
Nonenal	wax, citrus	0.03−0.47
Phenylacetaldehyde	green, grass, honey	0.05−4.5
**Alcohols**		
Phenylethyl alcohol	flowers, honey, pollen	0.01−2.26
Benzyl alcohol	roses, almond	0.05−0.13
**Terpenols**		
Camphor	mint	0−0.23
Borneol	pine tree	0−0.2
4-terpineol	spices, wood, soil	0.02−0.14
α-terpineol	flowers, lilac, sweet	0−0.2
**Lactones**		
γ-nonalactone	coconut, peach	0.03−0.11
**Fatty acids**		
Nonanoic acid	wax, dry, fatty	0.12−0.67
Vanillic acid	vanilla	0.07−0.86
Octanoic acid	coconut, lactic, rancid, cheese, sweat	0.14−3.38
Dodecanoic acid	coconut, fatty, metallic	0−6.3
Benceneacetic acid	honey, fruity, sour	0−3.0
**Furans**		
Furfural	caramel, candy	0−0.19

## Supplementary Materials

The following are available online at https://www.mdpi.com/2304-8158/9/2/133/s1. Table S1: Size of cork granulates (mm) used in this study (*n* = 20), Table S2: Individual aromatic composition in the studied granulates and corks at 3, 5, and 15 days of maceration.
