# Supplementary material for: Aromatic Potential and Bioactivity of Cork Stoppers and Cork By-Products
Source: Foods. 2020 Jan 28;9(2):133. doi: 10.3390/foods9020133 (PMC7073939; doi:10.3390/foods9020133)
Supplement: Supplementary file 1 [file foods-09-00133-s001.pdf]

Table S1. Size of cork granulates (mm) used in this study (n = 20).

CORK GRANULATES			
**A (high size)**			
6.3	10.4	7.2	9.1
11.7	10	9.2	6.9
12.7	14.3	8.3	10.8
6.1	11.7	7.7	7.7
8.6	10.4	6.9	7.7
**B (medium size)**			
3	1.6	3.4	2.2
3.1	5.9	3.8	2.3
3.8	2.4	2.7	1.6
5.3	2.9	2.7	2.7
2	3.7	4.4	3.7
**C (low size)**			
1.2	1.7	0.98	1.03
1.6	0.85	1.1	1.4
2	0.86	1.1	1.6
1.5	1.5	1.1	1.9
1.5	1.7	1.3	1.3

Table S2. Individual aromatic composition in the studied granulates and corks at 3, 5 and 15 days of maceration.

**MACERATION TIME**	**3 DAYS**											
	**GRANULATES**						**CORKS**					
	**A**		**B**		**C**		**A**		**B**		**C**	
	ẋ	SD (±)	ẋ	SD (±)	ẋ	SD (±)	ẋ	SD (±)	ẋ	SD (±)	ẋ	SD (±)
Vanillin	84.55 d	1.63	93.47 e	0.91	75.54 c	1.64	12.41 a	0.55	21.65 b	0.07	14.85 a	0.16
Acetovanillone	7.29 c	0.06	8.96 e	0.04	7.90 d	0.14	0.61 a	0.08	1.44 b	0.19	1.30 b	0.10
**Vainillins**	**91.84 d**	**1.11**	**102.44 e**	**0.61**	**83.44 c**	**1.06**	**13.02 a**	**0.34**	**23.09 b**	**0.08**	**16.14 a**	**0.04**
Guaicol	0.46 d	0.05	0.15 bc	0.02	0.17 c	0.01	0.12 abc	0.03	0.04 ab	0.01	0.03 a	0.00
4-vinylguaicol	7.71 b	0.18	9.27 c	0.28	6.98 b	0.26	1.02 a	0.11	0.80 a	0.18	0.44 a	0.18
Eugenol	0.09 bc	0.01	0.10 bc	0.00	0.14 c	0.04	0.03 ab	0.01	0.03 ab	0.01	0.01 a	0.01
Isoeugenol	0.30 ab	0.05	0.97 c	0.33	0.74 c	0.04	0.06 a	0.02	0.10 a	0.05	0.08 a	0.02
Cerulignol	0.76 b	0.15	1.54 c	0.13	0.22 a	0.02	0.04 a	0.00	0.12 a	0.03	0.09 a	0.04
**Volatile phenols**	**9.32 c**	**0.07**	**12.04 d**	**0.15**	**8.25 b**	**0.10**	**1.26 a**	**0.04**	**1.09 a**	**0.07**	**0.65 a**	0.02
Benzaldehyde	0.06 a	0.01	0.06 a	0.01	0.21 c	0.01	0.12 b	0.02	0.03 a	0.01	0.03 a	0.01
Nonennal	0.17 b	0.00	0.29 c	0.02	0.12 b	0.02	0.03 a	0.01	0.36 c	0.04	0.32 c	0.01
Phenylacetaldehyde	0.27 a	0.04	0.18 a	0.07	3.66 b	0.50	0.05 a	0.02	0.05 a	0.03	0.05 a	0.02
**Aldehydes**	**0.49 a**	**0.02**	**0.53 a**	**0.04**	**3.99 b**	**0.28**	**0.20 a**	**0.01**	**0.45 a**	**0.02**	**0.40 a**	**0.01**
Phenylethyl alcohol	0.14 ab	0.18	0.22 ab	0.02	0.34 b	0.05	0.01 a	0.01	0.03 a	0.01	0.03 a	0.01
Benzyl alcohol	0.08 a	0.01	0.12 a	0.01	0.05 a	0.02	0.10 a	0.01	0.13 a	0.06	0.07 a	0.05
**Alcohols**	**0.22 a**	**0.12**	**0.35 a**	**0.00**	**0.39 a**	**0.03**	**0.11 a**	**0.00**	**0.15 a**	**0.04**	**0.09 a**	**0.03**
Camphor	0.16 a	0.10	0.07 a	0.00	0.01 a	0.01	0.00 a	0.00	0.00 a	0.00	0.00 a	0.00

Borneol	0.11 b	0.04	0.05 ab	0.02	0.07 ab	0.04	0.00 a	0.00	0.00 a	0.00	0.00 a	0.00
4-terpineol	0.12 a	0.07	0.08 a	0.03	0.05 a	0.03	0.04 a	0.01	0.14 a	0.02	0.09 a	0.01
α-terpineol	0.06 a	0.01	0.06 a	0.01	0.20 b	0.04	0.01 a	0.01	0.03 a	0.00	0.02 a	0.02
**Terpenols**	**0.45 b**	**0.04**	**0.25 ab**	**0.01**	**0.33 ab**	**0.01**	**0.05 a**	**0.01**	**0.18 ab**	**0.01**	**0.11 ab**	**0.01**
ɣ-nonalactone	0.04 a	0.03	0.07 a	0.04	0.03 a	0.01	0.03 a	0.00	0.07 a	0.05	0.05 a	0.02
**Lactones**	**0.04 a**	**0.03**	**0.07 a**	**0.04**	**0.03 a**	**0.01**	**0.03 a**	**0.00**	**0.07 a**	**0.05**	**0.05 a**	**0.02**
Nonanoic acid	0.46 a	0.05	0.54 a	0.08	0.34 a	0.13	0.36 a	0.01	0.54 a	0.05	0.56 a	0.05
Vanillic acid	0.43 cd	0.05	0.58 d	0.07	0.32 bc	0.07	0.16 ab	0.05	0.14 ab	0.04	0.07 a	0.00
Octanoic acid	0.30 a	0.05	0.43 a	0.15	0.27 a	0.09	0.24 a	0.04	0.29 a	0.01	0.28 a	0.07
Dodecanoic acid	0.03 b	0.01	0.03 b	0.00	0.07 c	0.00	0.00 a	0.00	0.00 a	0.00	0.00 a	0.00
Benceneacetic acid	0.76 c	0.06	1.05 b	0.07	0.55 d	0.08	0.00 a	0.00	0.00 a	0.00	0.00 a	0.00
**Fatty acids**	**1.98 bc**	**0.02**	**2.63 c**	**0.05**	**1.54 ab**	**0.05**	**0.75 a**	**0.03**	**0.97 a**	**0.02**	**0.91 a**	0.01
Furfural	0.08 b	0.03	0.02 a	0.02	0.02 a	0.02	0.001 a	0.00	0.001 a	0.01	0.005 a	0.01
**Furans**	**0.08 b**	**0.03**	**0.02 a**	**0.02**	**0.02 a**	**0.02**	**0.001 a**	**0.00**	**0.001 a**	**0.01**	**0.005 a**	**0.01**
**Totals**	**104.42 b**	**0.38**	**118.33 c**	**0.20**	**97.98 b**	**0.36**	**15.43 a**	**0.12**	**26.00 a**	**0.03**	**18.36 a**	**0.02**

**MACERATION TIME**	**5 DAYS**											
	**GRANULATES**						**CORKS**					
	**A**		**B**		**C**		**A**		**B**		**C**	
	ẋ	SD (±)	ẋ	SD (±)	ẋ	SD (±)	ẋ	SD (±)	ẋ	SD (±)	ẋ	SD (±)
Vanillin	167.85 f	3.14	90.93 e	0.81	78.66 d	0.38	29.43 c	0.90	19.33 b	1.51	9.33 a	0.90
Acetovanillone	14.32 c	0.16	9.23 b	0.43	9.41 b	0.22	1.02 a	0.03	1.13 a	0.05	0.82 a	0.02
**Vainillins**	**182.16 f**	**2.10**	**100.16 e**	**0.27**	**88.08 d**	**0.12**	**30.46 c**	**0.61**	**20.47 b**	**1.03**	**10.15 a**	**0.63**
Guaicol	2.52 d	0.11	5.01 e	0.40	0.21 ab	0.03	0.85 bc	0.12	1.01 c	0.12	0.09 a	0.06

4-vinylguaicol	19.76 d	0.57	23.29 e	0.58	8.50 c	0.62	2.44 a	0.10	4.33 b	0.13	1.04 a	0.09
Eugenol	0.24 c	0.03	0.09 ab	0.04	0.16 bc	0.05	0.03 a	0.01	0.01 a	0.01	0.02 a	0.02
Isoeugenol	2.06 d	0.20	1.58 c	0.12	0.98 b	0.03	0.24 a	0.04	0.13 a	0.00	0.18 a	0.01
Cerulignol	2.18 d	0.02	1.32 c	0.07	0.42 b	0.11	0.24 ab	0.01	0.21 ab	0.01	0.15 a	0.03
**Volatile phenols**	**26.77 d**	**0.23**	**31.30 e**	**0.24**	**10.27 c**	**0.25**	**3.79 b**	**0.05**	**5.69 b**	**0.06**	**1.47 a**	**0.03**
Benzaldehyde	0.08 ab	0.03	0.06 a	0.01	0.13 b	0.01	0.03 a	0.01	0.02 a	0.02	0.02 a	0.01
Nonennal	0.47 b	0.04	0.29 a	0.01	0.15 a	0.02	0.54 b	0.08	0.25 a	0.02	0.20 a	0.05
Phenylacetaldehyde	0.46 a	0.06	0.28 a	0.03	2.75 b	0.50	0.13 a	0.06	0.13 a	0.05	0.08 a	0.03
**Aldehydes**	**1.01 a**	**0.01**	**0.63 a**	**0.01**	**3.03 b**	**0.28**	**0.70 a**	**0.04**	**0.40 a**	**0.02**	**0.31 a**	**0.02**
Phenylethyl alcohol	0.12 a	0.06	0.12 a	0.03	0.38 b	0.06	0.34 b	0.01	0.25 ab	0.02	0.79 c	0.02
Benzyl alcohol	0.13 a	0.01	0.11 a	0.02	0.06 a	0.00	0.09 a	0.01	0.08 a	0.02	0.06 a	0.04
**Alcohols**	**0.25 a**	**0.04**	**0.23 a**	**0.01**	**0.44 b**	**0.04**	**0.44 b**	**0.00**	**0.33 c**	**0.00**	**0.85 d**	**0.02**
Camphor	0.23 b	0.05	0.08 a	0.03	0.05 a	0.03	0.00 a	0.00	0.00 a	0.00	0.00 a	0.00
Borneol	0.20 b	0.05	0.08 a	0.03	0.04 a	0.01	0.00 a	0.00	0.00 a	0.00	0.00 a	0.00
4-terpineol	0.07 a	0.01	0.05 a	0.02	0.05 a	0.02	0.07 a	0.05	0.06 a	0.01	0.05 a	0.03
α-terpineol	0.12 c	0.01	0.08 bc	0.03	0.04 ab	0.01	0.002 a	0.00	0.003 a	0.00	0.002 a	0.00
**Terpenols**	**0.62 b**	**0.02**	**0.29 a**	**0.01**	**0.18 a**	**0.01**	**0.07 a**	**0.02**	**0.06 a**	**0.00**	**0.06 a**	**0.01**
ɣ-nonalactone	0.06 a	0.01	0.03 a	0.01	0.09 a	0.03	0.04 a	0.01	0.05 a	0.02	0.05 a	0.03
**Lactones**	**0.06 a**	**0.01**	**0.03 a**	**0.01**	**0.09 a**	**0.03**	**0.04 a**	**0.01**	**0.05 a**	**0.02**	**0.05 a**	**0.03**
Nonanoic acid	0.55 b	0.07	0.43 b	0.05	0.51 b	0.07	0.22 a	0.03	0.18 a	0.02	0.12 a	0.03
Vanillic acid	0.86 a	0.16	0.62 a	0.02	0.43 a	0.14	1.03 a	0.32	0.51 a	0.02	1.95 b	0.07
Octanoic acid	0.29 a	0.02	0.23 a	0.05	0.27 a	0.04	3.38 c	0.49	2.31 b	0.04	6.24 d	0.13
Dodecanoic acid	0.13 c	0.00	0.09 bc	0.02	0.04 ab	0.03	0.00 a	0.00	0.00 a	0.00	0.00 a	0.00
Benceneacetic acid	1.04 a	0.06	1.00 a	0.10	0.74 a	0.18	3.23 d	0.14	1.84 b	0.20	2.42 c	0.13

**Fatty acids**	**2.86 a**	**0.06**	**2.37 a**	**0.03**	**2.00 a**	**0.06**	**7.86 c**	**0.21**	**4.84 b**	**0.08**	**10.74 d**	**0.06**
Furfural	0.17 b	0.01	0.09 ab	0.02	0.02 ab	0.02	0.00 a	0.00	0.00 a	0.00	0.010 ab	0.09
**Furans**	**0.17 b**	**0.01**	**0.09 ab**	**0.02**	**0.02 ab**	**0.02**	**0.00 a**	**0.00**	**0.00 a**	**0.00**	**0.010 ab**	**0.09**
**Totals**	**213.92 c**	**0.73**	**135.09 b**	**0.11**	**104.10 b**	**0.11**	**43.35 a**	**0.21**	**31.84 a**	**0.36**	**23.72 a**	**0.21**

15 DAYS

**MACERATION TIME**	**GRANULATES**						**CORKS**					
	**A**		**B**		**C**		**A**		**B**		**C**	
	ẋ	SD (±)	ẋ	SD (±)	ẋ	SD (±)	ẋ	SD (±)	ẋ	SD (±)	ẋ	SD (±)
Vanillin	73.86 bc	1.37	79.84 c	2.96	71.53 b	0.39	32.01 a	1.85	29.95 a	0.22	27.35 a	0.06
Acetovanillone	5.32 b	0.13	8.42 c	0.21	13.78 d	0.36	1.92 a	0.11	1.85 a	0.02	2.35 a	0.08
**Vainillins**	**79.18 b**	**0.88**	**88.26 c**	**1.94**	**85.30 bc**	**0.02**	**33.93 a**	**1.23**	**31.80 a**	**0.13**	**29.70 a**	**0.01**
Guaicol	0.29 abc	0.06	0.46 c	0.09	0.41 bc	0.06	0.18 ab	0.02	0.11 a	0.07	0.08 a	0.03
4-vinylguaicol	4.10 c	0.14	11.12 d	0.16	10.91 d	0.30	1.41 a	0.10	2.33 b	0.05	1.31 a	0.06
Eugenol	0.15 abc	0.02	0.28 bc	0.07	0.29 c	0.10	0.08 ab	0.03	0.03 a	0.00	0.03 a	0.00
Isoeugenol	0.83 b	0.14	2.34 c	0.04	2.22 c	0.31	0.24 a	0.04	0.21 a	0.02	0.16 a	0.03
Cerulignol	0.31 b	0.04	0.68 c	0.07	0.80 c	0.10	0.11 ab	0.03	0.09 a	0.01	0.06 a	0.00
**Volatile phenols**	**5.67 c**	**0.06**	**14.89 d**	**0.05**	**14.63 d**	**0.12**	**2.02 ab**	**0.03**	**2.78 b**	**0.03**	**1.64 a**	**0.02**
Benzaldehyde	0.12 a	0.01	0.14 a	0.04	0.11 a	0.01	0.06 a	0.04	0.07 a	0.01	0.05 a	0.03
Nonennal	0.17 a	0.04	0.23 a	0.05	0.25 a	0.02	0.20 a	0.00	0.29 a	0.02	0.28 a	0.02
Phenylacetaldehyde	3.22 b	0.02	4.35 c	0.31	4.50 c	0.28	0.21 a	0.03	0.13 a	0.04	0.13 a	0.04
**Aldehydes**	**0.51 b**	**0.01**	**4.72 c**	**0.15**	**4.86 c**	**0.16**	**0.48 a**	**0.02**	**0.49 a**	**0.01**	**0.47 a**	**0.01**

Phenylethyl alcohol	0.26 a	0.03	0.61 ab	0.08	0.88 bc	0.13	1.13 c	0.04	2.22 d	0.17	2.26 d	0.18
Benzyl alcohol	0.12 a	0.07	0.10 a	0.04	0.11 a	0.04	0.06 a	0.04	0.06 a	0.03	0.07 a	0.05
**Alcohols**	**0.37 a**	**0.03**	**0.71 a**	**0.03**	**0.98 a**	**0.06**	**1.19 a**	**0.00**	**2.27 b**	**0.09**	**2.32 b**	**0.09**
Camphor	0.18 a	0.03	0.16 a	0.10	0.11 a	0.09	0.04 a	0.06	0.04 a	0.04	0.02 a	0.02
Borneol	0.14 a	0.09	0.13 a	0.05	0.11 a	0.04	0.04 a	0.01	0.02 a	0.01	0.02 a	0.02
4-terpineol	0.04 a	0.03	0.06 a	0.06	0.06 a	0.06	0.02 a	0.02	0.02 a	0.03	0.02 a	0.03
α-terpineol	0.07 a	0.00	0.09 a	0.01	0.11 a	0.03	0.04 a	0.06	0.01 a	0.02	0.01 a	0.01
**Terpenols**	**0.42 a**	**0.04**	**0.44 a**	**0.04**	**0.38 a**	**0.03**	**0.14 a**	**0.03**	**0.09 a**	**0.01**	**0.07 a**	**0.01**
ɣ-nonalactone	0.11 a	0.08	0.09 a	0.01	0.11 a	0.03	0.03 a	0.01	0.03 a	0.01	0.03 a	0.01
**Lactones**	**0.11 a**	**0.08**	**0.09 a**	**0.01**	**0.11 a**	**0.03**	**0.03 a**	**0.01**	**0.03 a**	**0.01**	**0.03 a**	**0.01**
Nonanoic acid	0.33 a	0.15	0.41 ab	0.08	0.67 b	0.09	0.44 ab	0.04	0.45 ab	0.03	0.42 ab	0.03
Vanillic acid	0.27 ab	0.04	0.39 bc	0.03	0.52 c	0.07	0.13 a	0.05	0.12 a	0.07	0.09 a	0.02
Octanoic acid	0.30 a	0.10	0.45 a	0.12	0.46 a	0.01	6.32 d	0.02	4.47 b	0.10	4.96 c	0.18
Dodecanoic acid	0.00 a	0.00	0.00 a	0.00	0.02 a	0.02	0.22 b	0.08	0.18 ab	0.07	0.14 ab	0.06
Benceneacetic acid	0.62 bc	0.16	1.69 d	0.10	0.99 c	0.20	0.14 a	0.02	0.18 ab	0.02	0.08 a	0.03
**Fatty acids**	**1.51 a**	**0.07**	**2.95 b**	**0.05**	**2.66 b**	**0.07**	**7.26 d**	**0.03**	**5.41 c**	**0.03**	**5.69 c**	**0.07**
Furfural	0.19 a	0.15	0.10 a	0.14	0.12 a	0.16	0.03 a	0.05	0.02 a	0.02	0.00 a	0.00
**Furans**	**0.19 a**	**0.15**	**0.10 a**	**0.14**	**0.12 a**	**0.16**	**0.03 a**	**0.05**	**0.02 a**	**0.02**	**0.00 a**	**0.00**
**Totals**	**88.08 b**	**0.29**	**112.17 c**	**0.67**	**109.06 c**	**0.06**	**45.07 a**	**0.43**	**42.90 a**	**0.05**	**39.93 a**	**0.04**
